# Crystal structure of the inter­metallic compound SrCdPt

**DOI:** 10.1107/S1600536814025823

**Published:** 2014-11-29

**Authors:** Fakhili Gulo, Jürgen Köhler

**Affiliations:** a Department of Chemical Education, Sriwijaya University, Inderalaya, Ogan Ilir 30662, South Sumatra, Indonesia; b Max Planck Institut für Festkörperforschung, Heisenbergstrasse 1, 70698 Stuttgart, Germany

**Keywords:** crystal structure, TiNiSi structure type, six-membered rings of strontium, inter­metallic compound

## Abstract

The crystal structure of the title compound, strontium cadmium platinum, adopts the TiNiSi structure type with the Sr atoms on the Ti, the Cd atoms on the Ni and the Pt atoms on the Si positions, respectively. The Pt atoms form cadmium-centred tetra­hedra that are condensed into a three-dimensional network with channels parallel to the *b*-axis direction in which the Sr atoms are located. The latter are bonded to each other in the form of six-membered rings with chair conformations. All atoms in the SrCdPt structure are situated on a mirror plane.

## Abstract 2

The title compound crystallizes in the TiNiSi structure type in the space group *Pnma*. St atoms are bonded to each other, forming six-membered rings with chair conformation whilst Pt atoms form zigzag chains of cadmium-centred tetra­hedra, building up the three-dimensional network.

## Chemical context   

Exploratory synthesis of polar inter­metallic phases has proven to be productive in terms of novel compositions, new and unprecedented structures, and unusual bonding regimes (Corbett, 2010 ▶). Platinum has participated significantly in the formation of ternary inter­metallic compounds. Together with indium, a number of platinum phases have been reported, for example BaPtIn_3_ (Palasyuk & Corbett, 2007 ▶), SrPtIn (Hoffmann & Pöttgen, 1999 ▶), CaPtIn_2_ (Hoffmann *et al.*, 1999 ▶) or Ca_2_Pt_2_In (Muts *et al.*, 2007 ▶). Some other ternary inter­metallic compounds of platinum with cadmium, *viz.* Ca_2_CdPt_2_ (Samal & Corbett, 2012 ▶), Ca_6_Pt_8_Cd_16_, (Ba/Sr)Cd_4_Pt_2_ (Samal *et al.*, 2013 ▶), Ca_6_Cd_11_Pt (Gulo *et al.*, 2013 ▶) and CaCdPt (Kersting *et al.*, 2013 ▶) have been isolated recently. They demonstrate the diversity of the structures types adopted. In this communication, we present the crystal structure of SrCdPt.

## Structural commentary   

SrCdPt crystallizes in the TiNiSi structure type. The titanium, nickel, and silicon sites are occupied by strontium, cadmium, and platinum, respectively, in the structure of the title compound. Although platinum and nickel are in the same group in the periodic table, the platinum in SrCdPt occupies the silicon site and not the nickel site because platinum is the most electronegative metal in this structure, just like silicon in TiNiSi. A count of 56 valence electrons per cell is found in SrCdPt [(Sr:2 + Cd:2 +Pt:10) × 4] whilst TiNiSi contains only 32 valence electrons per cell.

**Figure 1:**
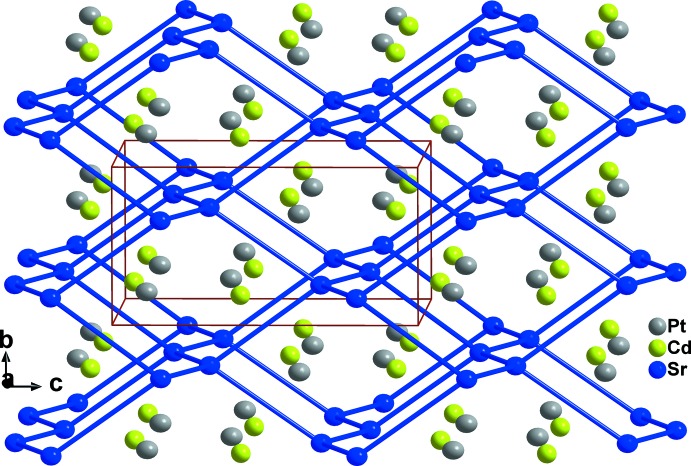
Projection of the crystal structure of SrCdPt approximately along [100]. Displacement ellipsoids are represented at the 90% probability level.

**Figure 2:**
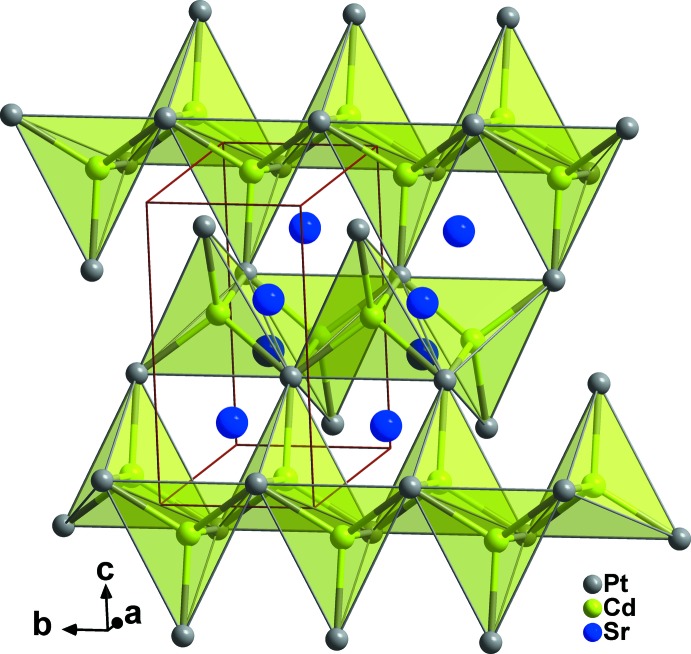
View of zigzag chains of cadmium-centred tetra­hedra of Pt atoms forming channels along the *b*-axis direction in the structure of SrCdPt.

In the compounds of the TiNiSi structure family, the metals listed first in the formula are linked to each other, forming six-membered rings in chair, half-chair, or boat conformations. The adopted conformation is not a function of the electron count, but is due to the nature of the respective metal (Landrum *et al.*, 1998 ▶). In the SrCdPt structure, the strontium atoms construct six-membered rings with chair conformations and Sr—Sr distances of 3.870 (2) Å, which is significantly shorter than the sum of the covalent radii of 4.30 Å (Emsley, 1999 ▶), indicating strong bonding inter­actions between them (Fig. 1 ▶). The existence of such strong Sr—Sr bonds is not noticeable in SrCd_4_Pt_2_ (Samal *et al.*, 2013 ▶). The platinum atoms in the structure of SrCdPt form zigzag chains of edge-sharing cadmium-centred tetra­hedra parallel to the *b*-axis direction. These chains are condensed *via* common corners with adjacent chains, building up the three-dimensional network with channels parallel to the *b*-axis direction in which the Sr atoms reside, as illustrated in Fig. 2 ▶.

**Figure 3:**
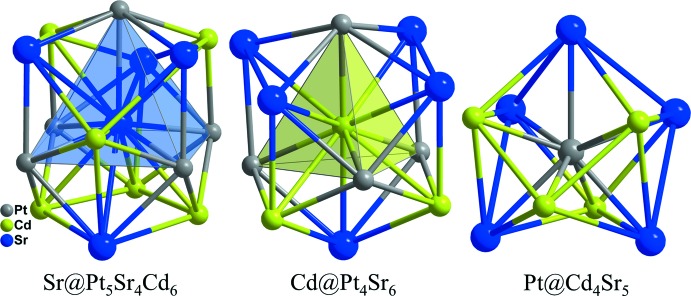
Coordination polyhedra of Sr, Cd, and Pt atoms in the structure of SrCdPt.

Strontium has an overall coordination number of 15 and is surrounded by four other strontium, six cadmium, and five platinum atoms. The Sr—Cd distances range from 3.3932 (13) to 3.6124 (17) Å, whereas the Sr—Pt distances vary only slightly, from 3.1943 (11) to 3.2238 (10) Å. Cadmium is located at a site that is surrounded by six strontium and four platinum atoms, whilst platinum has a coordination number of 9 defined by five strontium and four cadmium atoms. The environment of each atom in this structure is represented in Fig. 3 ▶. The inter­atomic distances (Sr—Cd, Sr—Pt, and Cd—Pt) are in good agreement with those found in the structures of some other ternary compounds in the alkaline earth–Cd–Pt system (Samal & Corbett, 2012 ▶; Samal *et al.*, 2013 ▶; Gulo *et al.*, 2013 ▶; Kersting *et al.*, 2013 ▶). In SrCdPt, the shortest Cd—Cd distance of 3.3197 (15) Å is too long to be considered as a bond. It is significantly longer than the sum of the covalent radii of 2.90 Å (Emsley, 1999 ▶). In contrast, cadmium atoms are bonded together, forming Cd_4_ tetra­hedra in SrCd_4_Pt_2_, Cd_8_ tetra­hedral stars in Ca_6_Cd_16_Pt_8_, and Cd_7_ penta­gonal bipyramids in Ca_6_Cd_11_Pt.

## Database survey   

A search of the *Pearson’s Crystal Data – Crystal Structure Database for Inorganic Compounds* (Villars & Cenzual, 2011 ▶) for the TiNiSi family of compounds returned 1101 entries with the same prototype. Two ternary compounds of them include strontium and platinum, one compound includes strontium with cadmium, and no compound had formed so far including both cadmium and platinum.

## Synthesis and crystallization   

Starting materials for the synthesis of the title compound were ingots of strontium (99.9+%, Alfa Aesar), cadmium powder (99.9+%, Alfa Aesar) and platinum powder (99.95%, Chempur). A stoichiometric mixture of these elements was weighed and loaded into a tantalum ampoule in an argon-filled dry box. The tantalum ampoule was then weld-sealed under an argon atmosphere and subsequently enclosed in an evacuated silica jacket. The sample was then heated to 1123 K for 15 h, followed by equilibration at 923 K for 4 days, and slow cooling to room temperature. The synthesis procedures were similar to general methods applied in some previous experiments (Gulo *et al.*, 2013 ▶).

## Refinement   

Crystal data, data collection and structure refinement details are summarized in Table 1 ▶. The highest remaining electron density is located 0.98 Å from the Pt site.

## Supplementary Material

Crystal structure: contains datablock(s) I. DOI: 10.1107/S1600536814025823/wm5093sup1.cif

Structure factors: contains datablock(s) I. DOI: 10.1107/S1600536814025823/wm5093Isup2.hkl

CCDC reference: 1036051

Additional supporting information:  crystallographic information; 3D view; checkCIF report

## Figures and Tables

**Table 1:** Experimental details

Crystal data	
Chemical formula	SrCdPt
*M*_r_	395.11
Crystal system, space group	Orthorhombic, *P* *n* *m* *a*
Temperature (K)	298
*a*, *b*, *c* ()	7.5748(15), 4.4774(9), 8.6383(17)
*V* (^3^)	292.97(10)
*Z*	4
Radiation type	Mo *K*
(mm^1^)	72.61
Crystal size (mm)	0.05 0.04 0.03

Data collection	
Diffractometer	Bruker *SMART* CCD
Absorption correction	Multi-scan (*SADABS*; Bruker, 2001 ▶)
*T*_min_, *T*_max_	0.043, 0.113
No. of measured, independent and observed [*I* > 2(*I*)] reflections	2231, 381, 338
*R*_int_	0.061
(sin /)_max_ (^1^)	0.664

Refinement	
*R*[*F* ^2^ > 2(*F* ^2^)], *wR*(*F* ^2^), *S*	0.030, 0.066, 1.07
No. of reflections	381
No. of parameters	19
_max_, _min_ (e ^3^)	2.22, 1.87

Computer programs: *SMART* and *SAINT* (Bruker, 2001 ▶), *SHELXS97* and *SHELXL97* (Sheldrick, 2008 ▶) and *DIAMOND* (Brandenburg, 2006 ▶).

## supplementary crystallographic information

### Crystal data

**Table:**

SrCdPt	*F*(000) = 656
*M**_r_* = 395.11	*D*_x_ = 8.958 Mg m^−^^3^
Orthorhombic, *P**n**m**a*	Mo *K*α radiation, λ = 0.71073 Å
Hall symbol: -P 2ac 2n	Cell parameters from 25 reflections
*a* = 7.5748 (15) Å	θ = 12–18°
*b* = 4.4774 (9) Å	µ = 72.61 mm^−^^1^
*c* = 8.6383 (17) Å	*T* = 298 K
*V* = 292.97 (10) Å^3^	Block, brown
*Z* = 4	0.05 × 0.04 × 0.03 mm

### Data collection

**Table:**

Bruker SMART CCD diffractometer	381 independent reflections
Radiation source: fine-focus sealed tube	338 reflections with *I* > 2σ(*I*)
Graphite monochromator	*R*_int_ = 0.061
Detector resolution: 0 pixels mm^-1^	θ_max_ = 28.1°, θ_min_ = 3.6°
ω scans	*h* = −9→9
Absorption correction: multi-scan (*SADABS*; Bruker, 2001)	*k* = −5→5
*T*_min_ = 0.043, *T*_max_ = 0.113	*l* = −11→11
2231 measured reflections	

### Refinement

**Table:**

Refinement on *F*^2^	0 restraints
Least-squares matrix: full	Primary atom site location: structure-invariant direct methods
*R*[*F*^2^ > 2σ(*F*^2^)] = 0.030	Secondary atom site location: difference Fourier map
*wR*(*F*^2^) = 0.066	*w* = 1/[σ^2^(*F*_o_^2^) + (0.0307*P*)^2^] where *P* = (*F*_o_^2^ + 2*F*_c_^2^)/3
*S* = 1.07	(Δ/σ)_max_ < 0.001
381 reflections	Δρ_max_ = 2.22 e Å^−^^3^
19 parameters	Δρ_min_ = −1.87 e Å^−^^3^

### Special details

**Table:**

Geometry. All e.s.d.'s (except the e.s.d. in the dihedral angle between two l.s. planes) are estimated using the full covariance matrix. The cell e.s.d.'s are taken into account individually in the estimation of e.s.d.'s in distances, angles and torsion angles; correlations between e.s.d.'s in cell parameters are only used when they are defined by crystal symmetry. An approximate (isotropic) treatment of cell e.s.d.'s is used for estimating e.s.d.'s involving l.s. planes.
Refinement. Refinement of *F*^2^ against ALL reflections. The weighted *R*-factor *wR* and goodness of fit *S* are based on *F*^2^, conventional *R*-factors *R* are based on *F*, with *F* set to zero for negative *F*^2^. The threshold expression of *F*^2^ > σ(*F*^2^) is used only for calculating *R*-factors(gt) *etc*. and is not relevant to the choice of reflections for refinement. *R*-factors based on *F*^2^ are statistically about twice as large as those based on *F*, and *R*- factors based on ALL data will be even larger.

### Fractional atomic coordinates and isotropic or equivalent isotropic displacement parameters (Å^2^)

**Table:**

	*x*	*y*	*z*	*U*_iso_*/*U*_eq_	
Pt	0.27016 (7)	0.2500	0.37717 (7)	0.0150 (2)	
Cd	0.14353 (12)	0.2500	0.06550 (12)	0.0140 (3)	
Sr	0.02883 (16)	0.2500	0.68094 (16)	0.0141 (3)	

### Atomic displacement parameters (Å^2^)

**Table:**

	*U*^11^	*U*^22^	*U*^33^	*U*^12^	*U*^13^	*U*^23^
Pt	0.0175 (3)	0.0114 (3)	0.0160 (4)	0.000	0.0005 (2)	0.000
Cd	0.0173 (6)	0.0120 (5)	0.0127 (6)	0.000	0.0013 (4)	0.000
Sr	0.0161 (7)	0.0116 (6)	0.0147 (7)	0.000	0.0006 (5)	0.000

### Geometric parameters (Å, º)

**Table:**

Pt—Cd^i^	2.8435 (8)	Cd—Sr^xi^	3.4336 (17)
Pt—Cd^ii^	2.8435 (8)	Cd—Sr^v^	3.4879 (13)
Pt—Cd	2.8581 (13)	Cd—Sr^iv^	3.4879 (13)
Pt—Cd^iii^	2.8713 (12)	Cd—Sr^iii^	3.6124 (17)
Pt—Sr^iv^	3.1943 (11)	Sr—Pt^i^	3.1943 (11)
Pt—Sr^v^	3.1943 (11)	Sr—Pt^ii^	3.1943 (11)
Pt—Sr	3.1980 (15)	Sr—Pt^vi^	3.2238 (10)
Pt—Sr^vi^	3.2238 (10)	Sr—Pt^vii^	3.2238 (10)
Pt—Sr^vii^	3.2238 (10)	Sr—Cd^vii^	3.3932 (13)
Cd—Pt^iv^	2.8435 (8)	Sr—Cd^vi^	3.3932 (13)
Cd—Pt^v^	2.8435 (8)	Sr—Cd^xii^	3.4336 (17)
Cd—Pt^viii^	2.8713 (12)	Sr—Cd^i^	3.4879 (13)
Cd—Cd^ix^	3.3197 (15)	Sr—Cd^ii^	3.4879 (13)
Cd—Cd^x^	3.3197 (15)	Sr—Cd^viii^	3.6124 (17)
Cd—Sr^vii^	3.3932 (13)	Sr—Sr^vi^	3.870 (2)
Cd—Sr^vi^	3.3932 (13)		
			
Cd^i^—Pt—Cd^ii^	103.87 (4)	Pt^viii^—Cd—Sr^iv^	130.66 (3)
Cd^i^—Pt—Cd	128.03 (2)	Cd^ix^—Cd—Sr^iv^	97.51 (2)
Cd^ii^—Pt—Cd	128.03 (2)	Cd^x^—Cd—Sr^iv^	175.11 (5)
Cd^i^—Pt—Cd^iii^	71.03 (3)	Sr^vii^—Cd—Sr^iv^	120.84 (3)
Cd^ii^—Pt—Cd^iii^	71.03 (3)	Sr^vi^—Cd—Sr^iv^	70.47 (2)
Cd—Pt—Cd^iii^	119.54 (3)	Sr^xi^—Cd—Sr^iv^	117.17 (3)
Cd^i^—Pt—Sr^iv^	138.23 (3)	Sr^v^—Cd—Sr^iv^	79.86 (4)
Cd^ii^—Pt—Sr^iv^	69.04 (3)	Pt^iv^—Cd—Sr^iii^	58.47 (2)
Cd—Pt—Sr^iv^	70.13 (3)	Pt^v^—Cd—Sr^iii^	58.47 (2)
Cd^iii^—Pt—Sr^iv^	67.79 (3)	Pt—Cd—Sr^iii^	106.50 (4)
Cd^i^—Pt—Sr^v^	69.04 (3)	Pt^viii^—Cd—Sr^iii^	153.82 (4)
Cd^ii^—Pt—Sr^v^	138.23 (3)	Cd^ix^—Cd—Sr^iii^	109.17 (4)
Cd—Pt—Sr^v^	70.13 (3)	Cd^x^—Cd—Sr^iii^	109.17 (4)
Cd^iii^—Pt—Sr^v^	67.79 (3)	Sr^vii^—Cd—Sr^iii^	133.73 (2)
Sr^iv^—Pt—Sr^v^	88.99 (4)	Sr^vi^—Cd—Sr^iii^	133.73 (2)
Cd^i^—Pt—Sr	70.24 (3)	Sr^xi^—Cd—Sr^iii^	68.55 (3)
Cd^ii^—Pt—Sr	70.24 (3)	Sr^v^—Cd—Sr^iii^	66.02 (3)
Cd—Pt—Sr	125.53 (4)	Sr^iv^—Cd—Sr^iii^	66.02 (3)
Cd^iii^—Pt—Sr	114.93 (4)	Pt^i^—Sr—Pt^ii^	88.99 (4)
Sr^iv^—Pt—Sr	135.06 (2)	Pt^i^—Sr—Pt	99.38 (3)
Sr^v^—Pt—Sr	135.06 (2)	Pt^ii^—Sr—Pt	99.38 (3)
Cd^i^—Pt—Sr^vi^	142.88 (3)	Pt^i^—Sr—Pt^vi^	154.71 (5)
Cd^ii^—Pt—Sr^vi^	72.78 (3)	Pt^ii^—Sr—Pt^vi^	86.033 (18)
Cd—Pt—Sr^vi^	67.51 (3)	Pt—Sr—Pt^vi^	105.89 (3)
Cd^iii^—Pt—Sr^vi^	135.959 (18)	Pt^i^—Sr—Pt^vii^	86.033 (18)
Sr^iv^—Pt—Sr^vi^	76.441 (19)	Pt^ii^—Sr—Pt^vii^	154.71 (5)
Sr^v^—Pt—Sr^vi^	137.64 (2)	Pt—Sr—Pt^vii^	105.89 (3)
Sr—Pt—Sr^vi^	74.11 (3)	Pt^vi^—Sr—Pt^vii^	87.96 (4)
Cd^i^—Pt—Sr^vii^	72.78 (3)	Pt^i^—Sr—Cd^vii^	51.57 (2)
Cd^ii^—Pt—Sr^vii^	142.88 (3)	Pt^ii^—Sr—Cd^vii^	107.65 (4)
Cd—Pt—Sr^vii^	67.51 (3)	Pt—Sr—Cd^vii^	138.55 (2)
Cd^iii^—Pt—Sr^vii^	135.959 (18)	Pt^vi^—Sr—Cd^vii^	106.76 (4)
Sr^iv^—Pt—Sr^vii^	137.64 (2)	Pt^vii^—Sr—Cd^vii^	51.10 (2)
Sr^v^—Pt—Sr^vii^	76.441 (19)	Pt^i^—Sr—Cd^vi^	107.65 (4)
Sr—Pt—Sr^vii^	74.11 (3)	Pt^ii^—Sr—Cd^vi^	51.57 (2)
Sr^vi^—Pt—Sr^vii^	87.96 (4)	Pt—Sr—Cd^vi^	138.55 (2)
Pt^iv^—Cd—Pt^v^	103.87 (4)	Pt^vi^—Sr—Cd^vi^	51.10 (2)
Pt^iv^—Cd—Pt	117.50 (2)	Pt^vii^—Sr—Cd^vi^	106.76 (4)
Pt^v^—Cd—Pt	117.50 (2)	Cd^vii^—Sr—Cd^vi^	82.56 (4)
Pt^iv^—Cd—Pt^viii^	108.97 (3)	Pt^i^—Sr—Cd^xii^	50.65 (2)
Pt^v^—Cd—Pt^viii^	108.97 (3)	Pt^ii^—Sr—Cd^xii^	50.65 (2)
Pt—Cd—Pt^viii^	99.68 (3)	Pt—Sr—Cd^xii^	130.48 (4)
Pt^iv^—Cd—Cd^ix^	54.88 (2)	Pt^vi^—Sr—Cd^xii^	109.17 (3)
Pt^v^—Cd—Cd^ix^	119.11 (5)	Pt^vii^—Sr—Cd^xii^	109.17 (3)
Pt—Cd—Cd^ix^	122.75 (4)	Cd^vii^—Sr—Cd^xii^	58.19 (3)
Pt^viii^—Cd—Cd^ix^	54.10 (3)	Cd^vi^—Sr—Cd^xii^	58.19 (3)
Pt^iv^—Cd—Cd^x^	119.11 (5)	Pt^i^—Sr—Cd^i^	50.41 (2)
Pt^v^—Cd—Cd^x^	54.88 (2)	Pt^ii^—Sr—Cd^i^	105.21 (4)
Pt—Cd—Cd^x^	122.75 (4)	Pt—Sr—Cd^i^	50.11 (2)
Pt^viii^—Cd—Cd^x^	54.10 (3)	Pt^vi^—Sr—Cd^i^	154.30 (5)
Cd^ix^—Cd—Cd^x^	84.81 (5)	Pt^vii^—Sr—Cd^i^	90.55 (2)
Pt^iv^—Cd—Sr^vii^	167.74 (3)	Cd^vii^—Sr—Cd^i^	92.00 (2)
Pt^v^—Cd—Sr^vii^	86.46 (2)	Cd^vi^—Sr—Cd^i^	151.86 (5)
Pt—Cd—Sr^vii^	61.38 (3)	Cd^xii^—Sr—Cd^i^	95.54 (3)
Pt^viii^—Cd—Sr^vii^	60.64 (3)	Pt^i^—Sr—Cd^ii^	105.21 (4)
Cd^ix^—Cd—Sr^vii^	114.39 (5)	Pt^ii^—Sr—Cd^ii^	50.41 (2)
Cd^x^—Cd—Sr^vii^	61.52 (3)	Pt—Sr—Cd^ii^	50.11 (2)
Pt^iv^—Cd—Sr^vi^	86.46 (2)	Pt^vi^—Sr—Cd^ii^	90.55 (2)
Pt^v^—Cd—Sr^vi^	167.74 (3)	Pt^vii^—Sr—Cd^ii^	154.30 (5)
Pt—Cd—Sr^vi^	61.38 (3)	Cd^vii^—Sr—Cd^ii^	151.86 (5)
Pt^viii^—Cd—Sr^vi^	60.64 (3)	Cd^vi^—Sr—Cd^ii^	92.00 (2)
Cd^ix^—Cd—Sr^vi^	61.52 (3)	Cd^xii^—Sr—Cd^ii^	95.54 (3)
Cd^x^—Cd—Sr^vi^	114.39 (5)	Cd^i^—Sr—Cd^ii^	79.86 (4)
Sr^vii^—Cd—Sr^vi^	82.56 (4)	Pt^i^—Sr—Cd^viii^	134.25 (2)
Pt^iv^—Cd—Sr^xi^	60.31 (2)	Pt^ii^—Sr—Cd^viii^	134.25 (2)
Pt^v^—Cd—Sr^xi^	60.31 (2)	Pt—Sr—Cd^viii^	88.75 (4)
Pt—Cd—Sr^xi^	175.05 (4)	Pt^vi^—Sr—Cd^viii^	48.75 (2)
Pt^viii^—Cd—Sr^xi^	85.27 (3)	Pt^vii^—Sr—Cd^viii^	48.75 (2)
Cd^ix^—Cd—Sr^xi^	60.30 (3)	Cd^vii^—Sr—Cd^viii^	93.99 (3)
Cd^x^—Cd—Sr^xi^	60.30 (3)	Cd^vi^—Sr—Cd^viii^	93.99 (3)
Sr^vii^—Cd—Sr^xi^	121.81 (3)	Cd^xii^—Sr—Cd^viii^	140.77 (5)
Sr^vi^—Cd—Sr^xi^	121.81 (3)	Cd^i^—Sr—Cd^viii^	113.98 (3)
Pt^iv^—Cd—Sr^v^	120.35 (4)	Cd^ii^—Sr—Cd^viii^	113.98 (3)
Pt^v^—Cd—Sr^v^	59.65 (2)	Pt^i^—Sr—Sr^vi^	152.61 (6)
Pt—Cd—Sr^v^	59.46 (3)	Pt^ii^—Sr—Sr^vi^	94.42 (2)
Pt^viii^—Cd—Sr^v^	130.66 (3)	Pt—Sr—Sr^vi^	53.25 (3)
Cd^ix^—Cd—Sr^v^	175.11 (5)	Pt^vi^—Sr—Sr^vi^	52.64 (2)
Cd^x^—Cd—Sr^v^	97.51 (2)	Pt^vii^—Sr—Sr^vi^	101.34 (5)
Sr^vii^—Cd—Sr^v^	70.47 (2)	Cd^vii^—Sr—Sr^vi^	149.28 (6)
Sr^vi^—Cd—Sr^v^	120.84 (3)	Cd^vi^—Sr—Sr^vi^	95.53 (3)
Sr^xi^—Cd—Sr^v^	117.17 (3)	Cd^xii^—Sr—Sr^vi^	144.11 (3)
Pt^iv^—Cd—Sr^iv^	59.65 (2)	Cd^i^—Sr—Sr^vi^	102.75 (5)
Pt^v^—Cd—Sr^iv^	120.35 (4)	Cd^ii^—Sr—Sr^vi^	58.53 (3)
Pt—Cd—Sr^iv^	59.46 (3)	Cd^viii^—Sr—Sr^vi^	55.44 (3)

Symmetry codes: (i) −*x*+1/2, −*y*+1, *z*+1/2; (ii) −*x*+1/2, −*y*, *z*+1/2; (iii) *x*+1/2, *y*, −*z*+1/2; (iv) −*x*+1/2, −*y*, *z*−1/2; (v) −*x*+1/2, −*y*+1, *z*−1/2; (vi) −*x*, −*y*, −*z*+1; (vii) −*x*, −*y*+1, −*z*+1; (viii) *x*−1/2, *y*, −*z*+1/2; (ix) −*x*, −*y*, −*z*; (x) −*x*, −*y*+1, −*z*; (xi) *x*, *y*, *z*−1; (xii) *x*, *y*, *z*+1.
